# Supplementary material for: MCM8 interacts with DDX5 to promote R-loop resolution
Source: EMBO J. 2024 Jun 10;43(14):3044–71. doi: 10.1038/s44318-024-00134-0 (PMC11251167; doi:10.1038/s44318-024-00134-0)
Supplement: Supplementary file 1 — Appendix [file 44318_2024_134_MOESM1_ESM.pdf]

Supplementary Materials for

MCM8 interacts with DDX5 to promote R-loop resolution

Canxin Wen *et al*

*Corresponding author. Yingying Qin, qinyingying@sdu.edu.cn;

Yajuan Yang, YangYJ0204@sdu.edu.cn;

Shidou Zhao, shidouzhao@sdu.edu.cn

This PDF file includes:

Appendix Figures S1 to S3

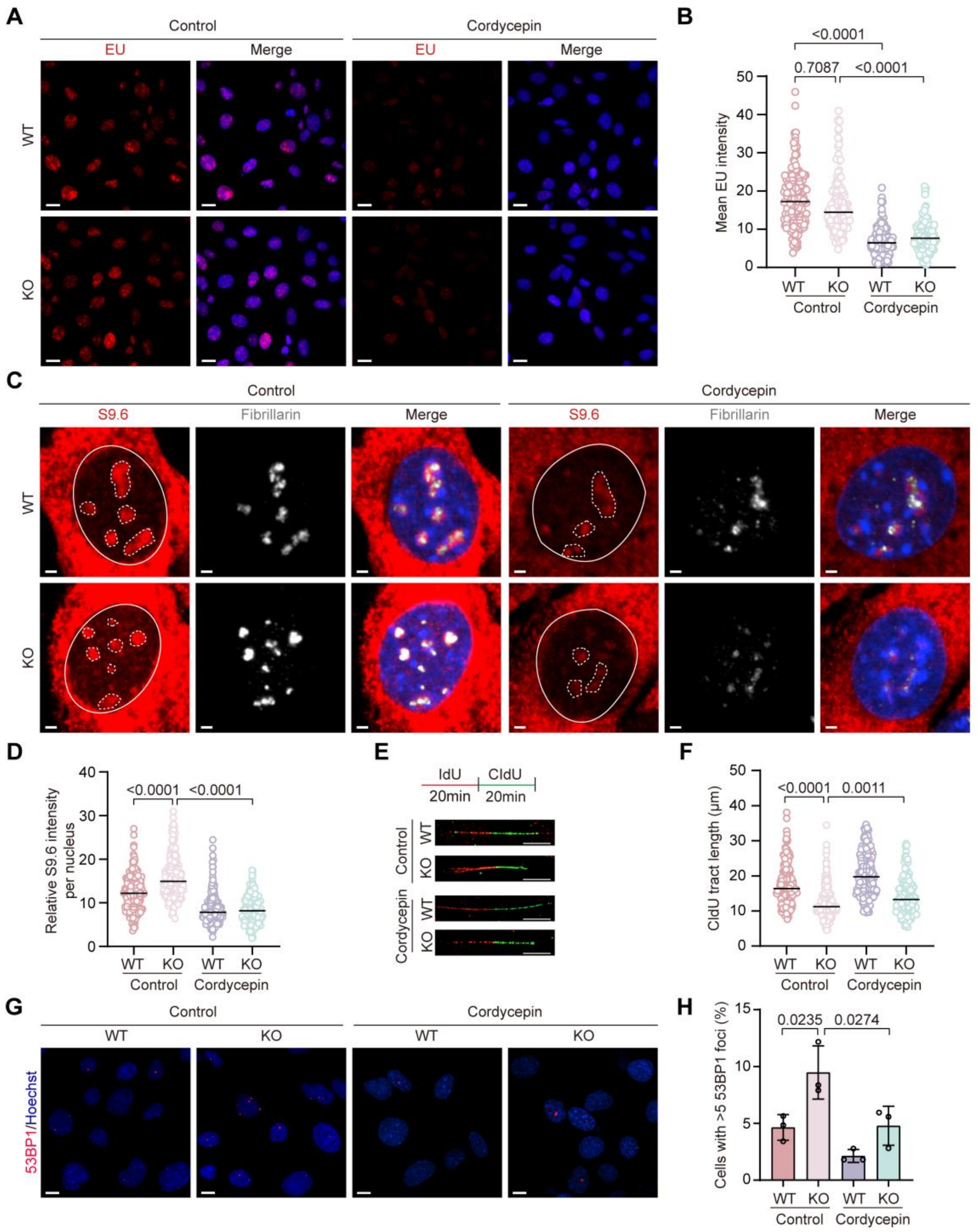

Appendix Fig S1 - Inhibiting R-loop formation by cordycepin treatment improved DNA damage in KO MEFs.

A Representative images of EU incorporation in WT and KO MEFs after treatment with cordycepin or its vehicle (dimethyl sulfoxide, DMSO) as a control. Scale bars: 20 μm.

B Quantification of mean EU signal intensity. At least 150 cells were scored per group. Three independent experiments were conducted.

C Representative images of S9.6 immunostaining to evaluate the R-loop level.

Nucleoli were shown by fibrillarin staining. Scale bars: 2 μm.

D Quantification of S9.6 nuclear intensity that was exclusive of nucleolar signal circled with the broken lines. At least 150 cells were scored per group. Three independent experiments were conducted.

E Immunostaining of IdU and CldU after the DNA fiber assay to evaluate RF speed. Scale bars: 5 μm.

F Quantification of the CldU tract length. At least 150 DNA fibers were scored per group. Three independent experiments were conducted.

G Representative images of 53BP1 immunostaining to evaluate the DNA damage level. Scale bars:10 μm.

H Percentage of cells containing more than five 53BP1 foci. At least 150 cells were scored per group. n = 3 independent replicates.

Data information: In (B, D, F), data are presented as the median with IQR, and in (H), data are presented as the mean ± SD. The statistical significance of the difference was analyzed by Kruskal-Wallis test followed by Dunn's multiple comparison test (B, D, F), and one-way ANOVA followed by Tukey's multiple comparison test (H), and the *P*-values were shown.

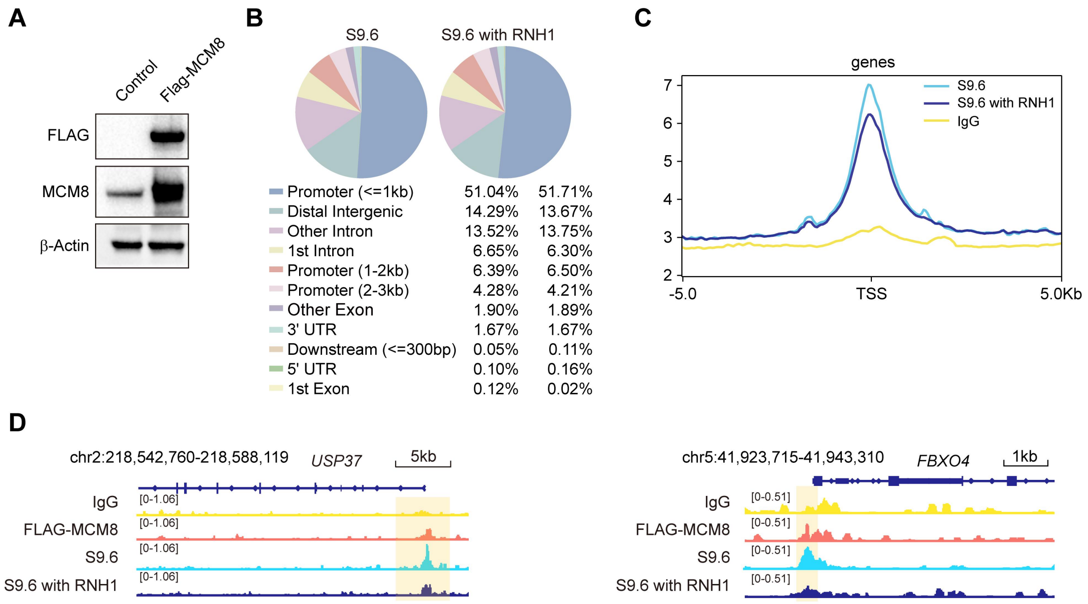

Appendix Fig S2 - Overexpression of RNH 1 mitigated R-loop accumulations.

A Confirmation of FLAG-MCM8 overexpressed in HEK293 cells after plasmid transfection by western blot. β-Actin was used as the loading control.

B The genomic distribution of R-loop CUT& Tag peaks in the S9.6 group and S9.6 with RNH1 group (with RNH1 overexpression). UTR, untranslated region.

C Genomic metaplots of genes with R-loop signals across the 5 kb window around TSS in the IgG, S9.6 group and S9.6 with RNH1 group. TSS, transcription start site.

D Snapshots of R-loop signals of the representative genes *USP37* and *FBXO4* by genome browser tracks in HEK293 cells with or without RNH1 overexpression. IgG (yellow), FLAG-MCM8 (orange), S9.6 (light blue) and S9.6 with RNH1 (dark blue) CUT& Tag data were shown.

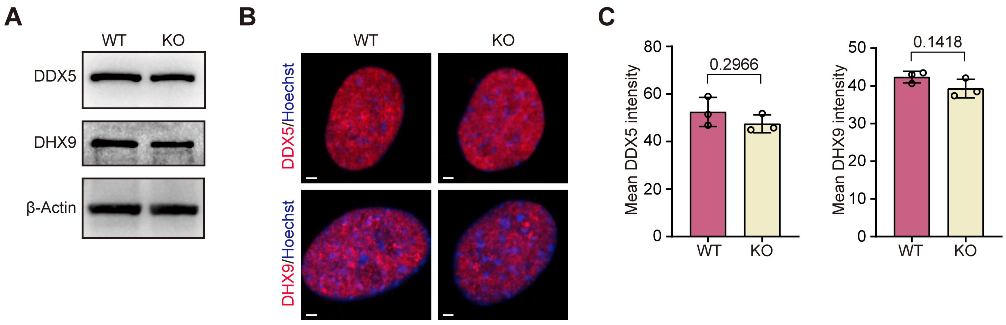

Appendix Fig S3 - The expression of DDX5 and DHX9 was not obviously affected after MCM8 deficiency.

A Detection of DDX5 and DHX9 expression in WT and KO MEFs by western blot. β-Actin was used as the loading control.

B Immunostaining of DDX5 and DHX9 in WT and KO MEFs. Scale bar: 2 μm.

C Quantification of mean DDX5 and DHX9 signal intensity in WT and KO MEFs. n = 3/3 replicates. Data are presented as the mean ± SEM, and the dots indicate individual replicates. The statistical significance of the difference was analyzed by two-tailed Mann-Whitney U test. The *P*-values were shown.
