# Supplementary material for: MCM8 interacts with DDX5 to promote R-loop resolution
Source: EMBO J. 2024 Jun 10;43(14):3044–71. doi: 10.1038/s44318-024-00134-0 (PMC11251167; doi:10.1038/s44318-024-00134-0)
Supplement: Supplementary file 14 — Expanded View Figures [file 44318_2024_134_MOESM14_ESM.pdf]

Expanded View Figures

Figure EV1. MCM8 deficiency caused germ cell depletion.

(**A**) Top: Schematic diagram of the *Mcm8* gene targeting strategy, in which a 1479 bp segment including Exon 7 of the *Mcm8* gene was deleted. The black scissors refer to the targeted gRNA regions and the arrows indicate the primers for genotyping. Bottom: Schematic diagram of the wild-type MCM8 protein and the predicted mutant protein in $Mcm8^{-/-}$ mice. MCM domain: minichromosome maintenance domain. (**B**) Genotyping results of the $Mcm8^{+/+}$, $Mcm8^{+/-}$, and $Mcm8^{-/-}$ mice. (**C**) Expression validation of MCM8 in the testes of the adult $Mcm8^{+/+}$ and $Mcm8^{-/-}$ mice by western blot. β-Actin was used as the loading control. (**D**) Representative images of $Mcm8^{+/+}$ and $Mcm8^{-/-}$ mice at 3 months. (**E**) Quantification of body weight to observe the growth of $Mcm8^{+/+}$ and $Mcm8^{-/-}$ mice at 3 months. $n = 10/10/10/10$. (**F**) The genotype and sex ratios of the offspring generated by mating $Mcm8^{+/-}$ male and female mice. $n = 185$ pups. (**G**) Cumulative pup number from 5 months of mating female or male $Mcm8^{+/+}$/$Mcm8^{-/-}$ mice with WT mice, respectively. $n = 6/6/6/6$. (**H**) Gross morphology of ovaries (left) and the ovary/body weight ratio (right) in adult $Mcm8^{+/+}$ and $Mcm8^{-/-}$ females. Scale bar, 1 mm. $n = 9/9$. (**I**) The gross morphology of the testes (left) and the testis/body weight ratio (right) in adult $Mcm8^{+/+}$ and $Mcm8^{-/-}$ males. Scale bar, 1 mm. $n = 9/9$. (**J**) Hematoxylin and eosin staining of $Mcm8^{+/+}$ and $Mcm8^{-/-}$ paraffin sections of ovaries from PD3, PD21, and 3 M females. 3 M, 3-month-old. Scale bars, 100 μm. (**K**) Hematoxylin and eosin staining of $Mcm8^{+/+}$ and $Mcm8^{-/-}$ paraffin sections of testes and epididymis from PD3 and 3 M males. 3 M, 3-month-old. Scale bars: 50 μm. (**L**) Immunostaining of STELLA (a marker for PGCs) in the genital ridges from WT, $Mcm8^{-/-}$, $Fancd2^{K559R/K559R}$, and $Mcm8^{-/-}Fancd2^{K559R/K559R}$ embryos and qualification of PGC numbers from E11.5 embryos with the indicated genotypes. Scale bars: 50 μm. $n = 5/5/5/5$ embryos. Data information: In (**E**, **H**, **I**, **L**), data are presented as the mean ± SD, and the dots indicate individual mice. The statistical significance of the difference was analyzed by unpaired two-tailed Student's *t*-test (**E**, **H**, **I**) and with one-way ANOVA followed by Dunnett's multiple comparison test (**L**), and the *P*-values were shown. Source data are available online for this figure.

© The Author(s)

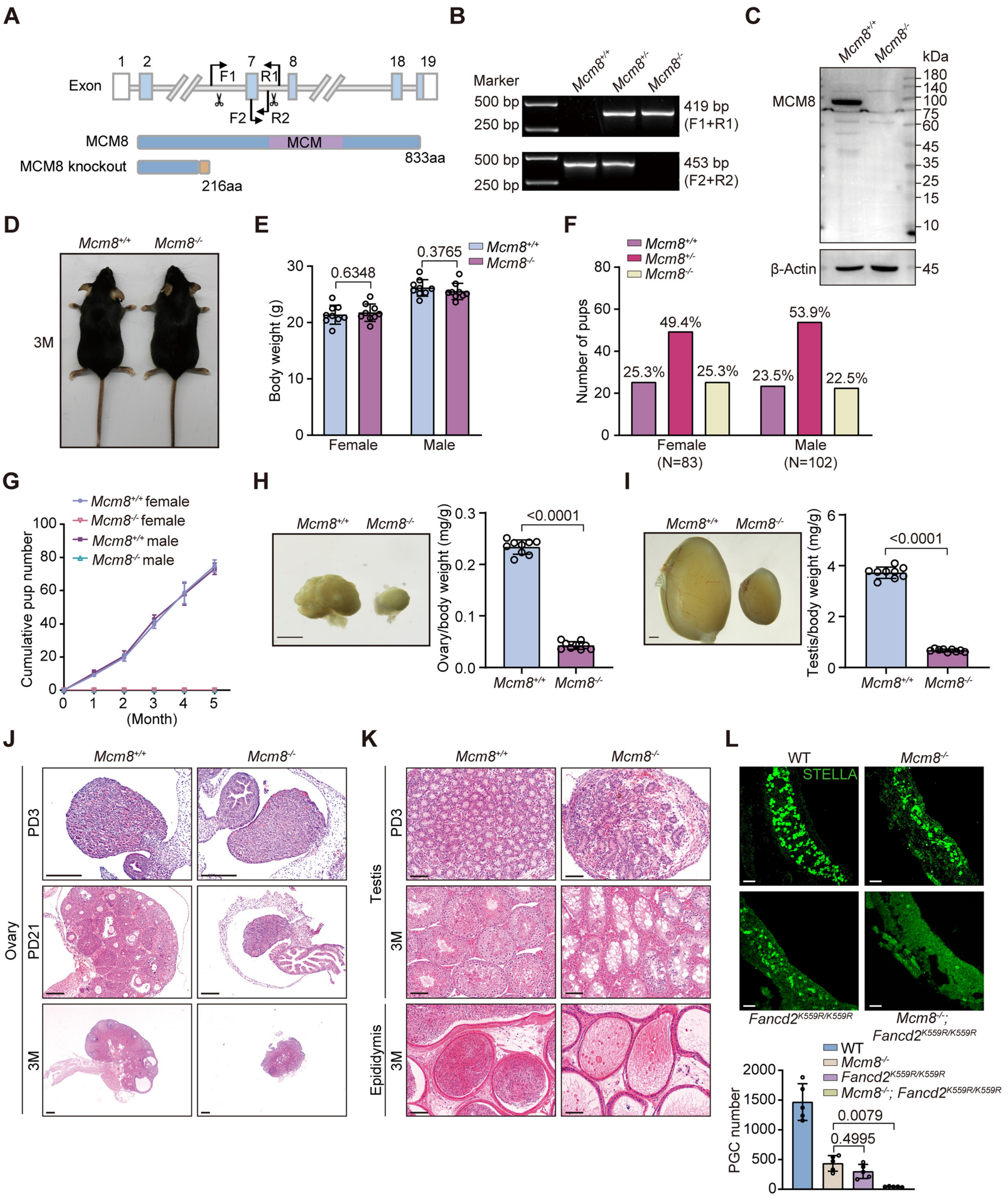
A
Exon
1
2
7
8
18
19
F1
R1
F2
R2
MCM8
MCM
833aa
MCM8 knockout
216aa
B
Marker
Mcm8+/+
Mcm8+/-
Mcm8-/-
500 bp
250 bp
419 bp (F1+R1)
453 bp (F2+R2)
C
Mcm8+/+
Mcm8-/-
kDa
MCM8
β-Actin
180
140
100
75
60
45
35
25
15
10
45
D
Mcm8+/+
Mcm8-/-
3M
E
Body weight (g)
0.6348
0.3765
Female
Male
F
Mcm8+/+
Mcm8+/-
Mcm8-/-
Number of pups
25.3%
49.4%
25.3%
23.5%
53.9%
22.5%
Female (N=83)
Male (N=102)
G
Mcm8+/+ female
Mcm8-/- female
Mcm8+/+ male
Mcm8-/- male
Cumulative pup number
(Month)
H
Ovary/body weight (mg/g)
<0.0001
I
Testis/body weight (mg/g)
<0.0001
J
Ovary
PD3
PD21
3M
K
Testis
Epididymis
L
WT
STELLA
Fancd2K559R/K559R
Mcm8-/-; Fancd2K559R/K559R
PGC number
0.0079
0.4995

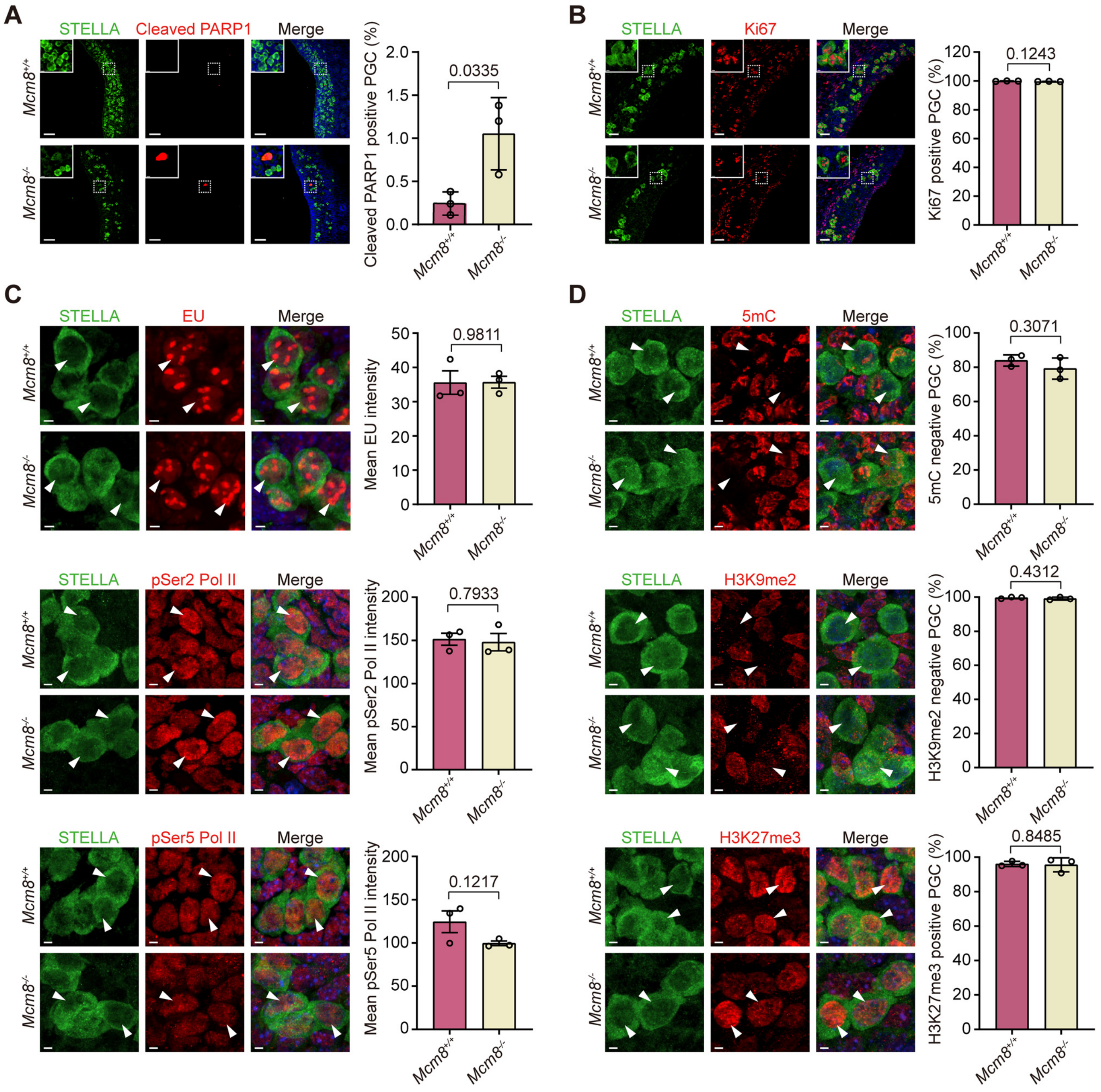

Figure EV2. Primordial germ cells undergone transcription activation and epigenetic reprogramming in *Mcm8*$^{-/-}$ embryos.

(**A**) Representative images of cleaved PARP1 immunostaining and percentage of the apoptotic PGCs (cleaved PARP1 positive) in E11.5 *Mcm8*$^{+/+}$ and *Mcm8*$^{-/-}$ genital ridges. Scale bars: 50 µm. Scale bars: 7 µm in the enlarged images. $n = 3/3$ embryos. (**B**) Representative images of Ki67 immunofluorescence staining and percentage of the PGCs actively progressing through the cell cycle (Ki67 positive) in E11.5 *Mcm8*$^{+/+}$ and *Mcm8*$^{-/-}$ genital ridges. Scale bars: 20 µm. Scale bars: 3 µm in the enlarged images. $n = 3/3$ embryos. (**C**) Representative images of EU incorporation and the phosphorylation of Ser2 and Ser5 within the C-terminal domain (CTD) of RNA polymerase II (Pol II) immunostaining in E11.5 *Mcm8*$^{+/+}$ and *Mcm8*$^{-/-}$ genital ridges to observe transcriptional output. Quantification of mean EU, pSer2 Pol II and pSer5 Pol II signal intensity in PGCs. Scale bars: 3 µm. $n = 3/3$ embryos. (**D**) Representative images of 5mC, H3K9me2 and H3K27me3 immunostaining in E11.5 *Mcm8*$^{+/+}$ and *Mcm8*$^{-/-}$ genital ridges to observe epigenetic modification. Quantification of percentage of the PGCs stained negative for 5mC and H3K9me2, and the PGCs stained positive for H3K27me3. Scale bars: 3 µm. $n = 3/3$ embryos. Data information: STELLA positivity indicates PGCs. In (**A**, **B**, **D**), data are presented as the mean ± SD, and in (**C**), data are presented as the mean ± SEM, and the dots indicate individual embryos. Dotted lines indicate positions of the enlarged images (**A**, **B**). Arrowheads indicate representative cells (**C**, **D**). The statistical significance of the difference was analyzed by unpaired two-tailed Student's *t*-test (**A–D**), and the *P*-values were shown. Source data are available online for this figure.

© The Author(s)

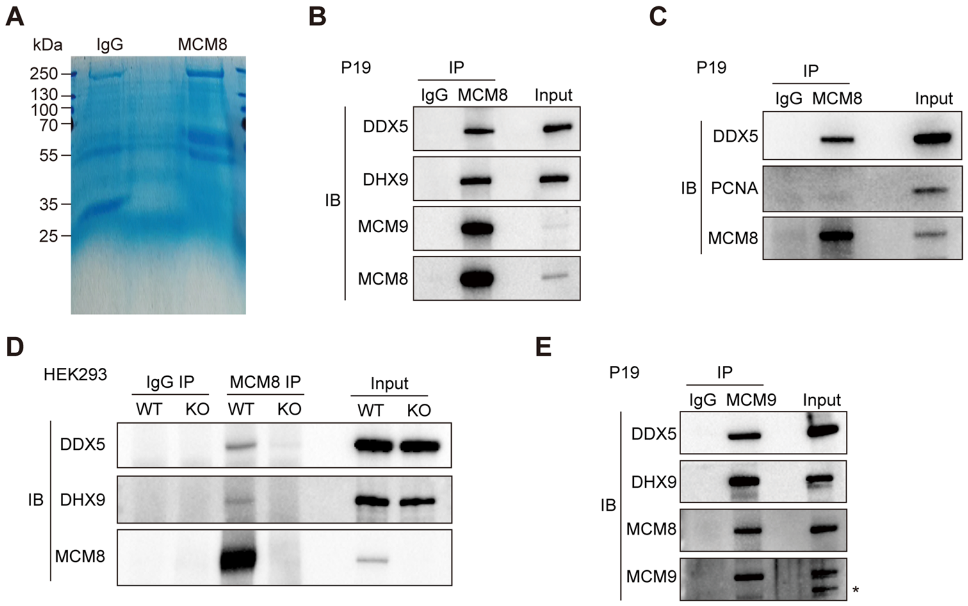

Figure EV3. MCM8 and MCM9 specifically interacted with DDX5 and DHX9.

(**A**) Representative image of 10% SDS-PAGE gel stained with Coomassie blue. MCM8, MCM8 antibody precipitated proteins in P19 cells; IgG, IgG antibody precipitated proteins in P19 cells. (**B**) Co-immunoprecipitation of endogenous MCM8 from P19 cell lysates, followed by immunoblot of DDX5, DHX9, MCM9 and MCM8. IgG was used as a negative control. (**C**) Co-immunoprecipitation of endogenous MCM8 from P19 cell lysates, followed by immunoblot of PCNA, DDX5 and MCM8. IgG was used as a negative control. (**D**) Co-immunoprecipitation of endogenous MCM8 from WT or KO HEK293 cell lysates, followed by immunoblot of DDX5, DHX9 and MCM8. IgG was used as a negative control. (**E**) Co-immunoprecipitation of endogenous MCM9 from P19 cell lysates, followed by immunoblot of DDX5, DHX9, MCM8 and MCM9. IgG was used as a negative control. Asterisk (*) indicated a non-specific band detected by anti-MCM9 antibody. Source data are available online for this figure.

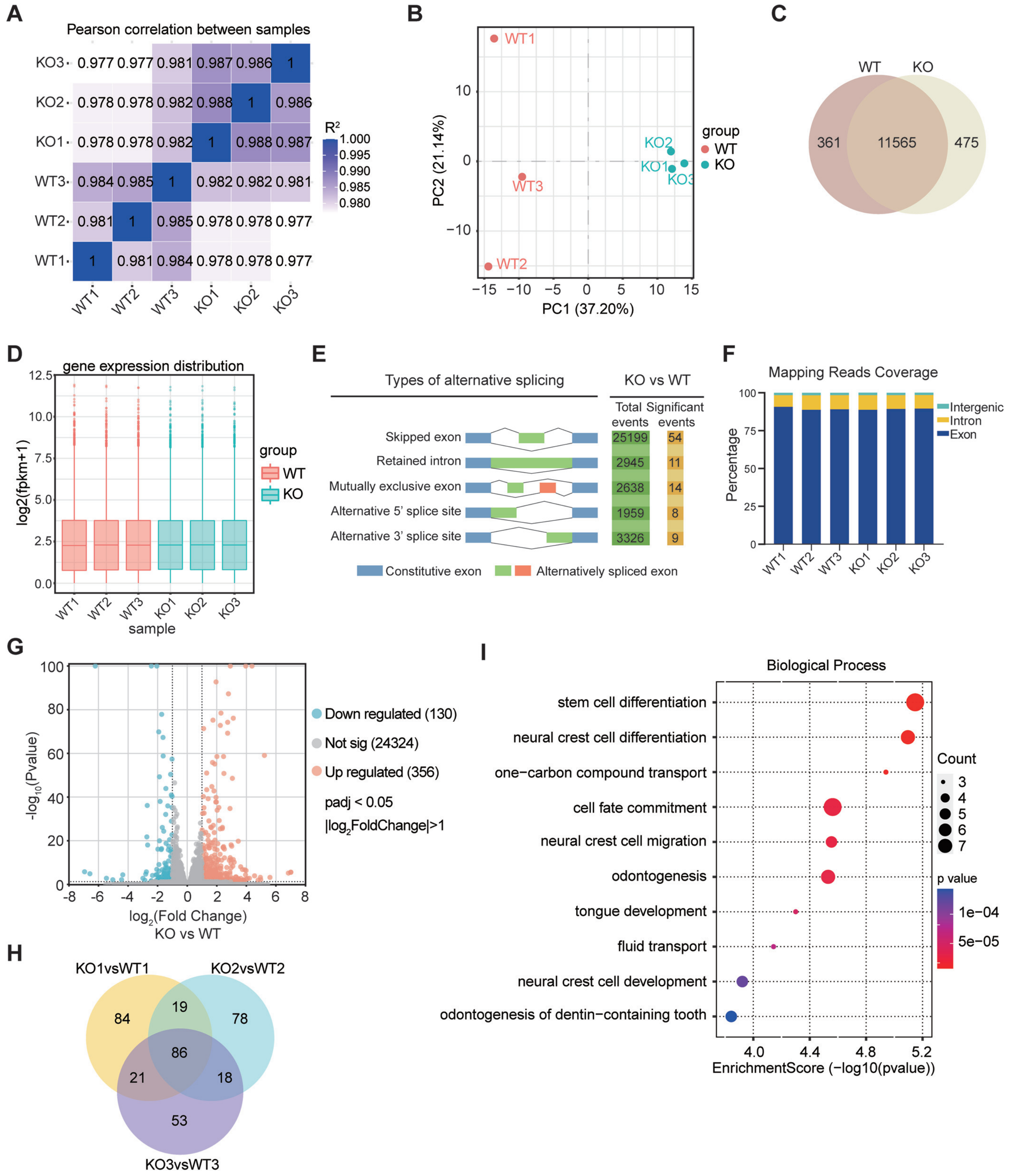
A
Pearson correlation between samples
KO3 0.977 0.977 0.981 0.987 0.986 1
KO2 0.978 0.978 0.982 0.988 1 0.986
KO1 0.978 0.978 0.982 1 0.988 0.987
WT3 0.984 0.985 1 0.982 0.982 0.981
WT2 0.981 1 0.985 0.978 0.978 0.977
WT1 1 0.981 0.984 0.978 0.978 0.977
WT1 WT2 WT3 KO1 KO2 KO3
R2
1.000
0.995
0.990
0.985
0.980
B
PC2 (21.14%)
PC1 (37.20%)
WT1
WT2
WT3
KO1
KO2
KO3
group
WT
KO
C
WT
KO
361
11565
475
D
gene expression distribution
log2(fpkm+1)
sample
group
WT
KO
E
Types of alternative splicing
KO vs WT
Total events
Significant events
Skipped exon 25199 54
Retained intron 2945 11
Mutually exclusive exon 2638 14
Alternative 5' splice site 1959 8
Alternative 3' splice site 3326 9
Constitutive exon
Alternatively spliced exon
F
Mapping Reads Coverage
Percentage
Intergenic
Intron
Exon
G
-log10(Pvalue)
log2(Fold Change)
KO vs WT
Down regulated (130)
Not sig (24324)
Up regulated (356)
padj < 0.05
|log2FoldChange|>1
H
KO1vsWT1
KO2vsWT2
KO3vsWT3
84
19
78
86
21
18
53
I
Biological Process
stem cell differentiation
neural crest cell differentiation
one−carbon compound transport
cell fate commitment
neural crest cell migration
odontogenesis
tongue development
fluid transport
neural crest cell development
odontogenesis of dentin−containing tooth
EnrichmentScore (−log10(pvalue))
Count
3
4
5
6
7
p value
1e−04
5e−05

 © The Author(s)

Figure EV4. Global transcriptional activity and alternative splicing events in *Mcm8* KO MEFs were not significantly changed.

(**A**) Pearson correlation coefficient analysis showing positive correlations between WT and KO groups. $n = 3/3$. (**B**) Principal component analysis (PCA) of RNA transcriptomes from WT and KO MEFs. (**C**) Venn diagram of the co-expressed genes between the WT and KO MEFs (fpkm>1). (**D**) Gene expression distribution of each sample in WT and KO group. (**E**) Schematic diagram of alternative splicing (AS) types and summary of AS analysis performed in WT and KO group. The numbers of total and different AS events in each category upon MCM8 deletion are indicated. FDR < 0.05 were considered significant. (**F**) Mapping reads coverage of each sample in WT and KO group. The percentages of reads aligned to exon, intron, and intergenic regions were shown. (**G**) Volcano plot of differentially expressed genes (DEGs) from RNA-seq in KO group compared to WT group (fpkm>0). |log2FC|>1 and an adjusted p value < 0.05 were considered significant. (**H**) Venn diagram showing 86 genes common to the DEGs from each KO sample compared to WT controls. (**I**) GO enrichment analysis of biological processes for the 86 genes showing the top 10 enriched terms. Source data are available online for this figure.

© The Author(s)

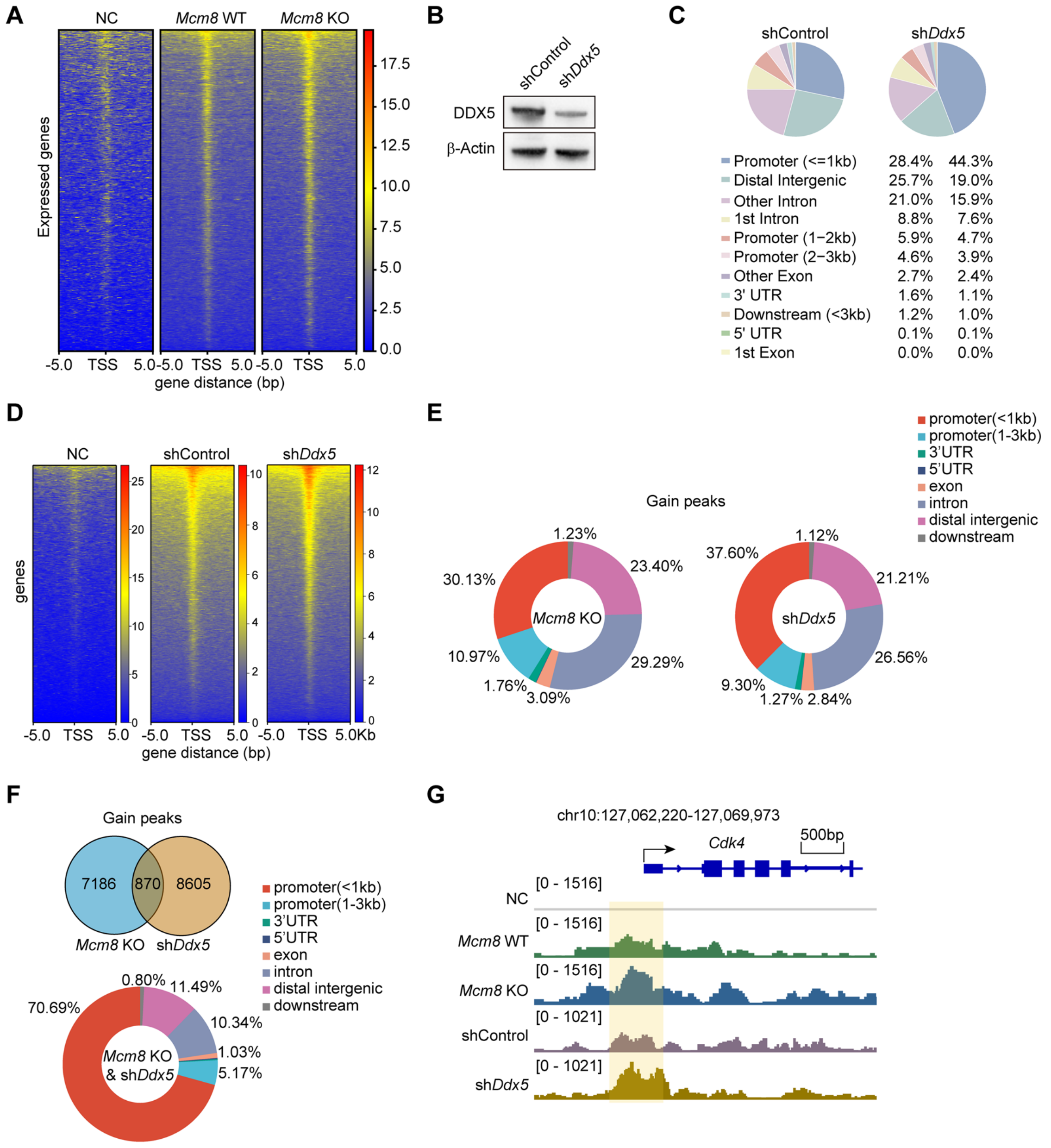

Figure EV5. R-loop gain peaks were overlapped in DDX5-knockdown and MCM8-deficient cells.

(**A**) Heatmaps showing the distribution of the R-loop signal of all expressed genes (rpkm>1) in WT and KO MEFs. R-loop signal was sorted based on gene expression level obtained from the RNA sequencing analysis of MEFs from high to low. The region −5 kb/+5 kb around the TSS is individually displayed. TSS, transcription start site. (**B**) Confirmation of DDX5 knockdown in MEFs using sh*Ddx5* adenovirus by western blot. β-Actin was used as the loading control. (**C**) The genomic distribution of R-loop CUT&Tag peaks in shControl MEFs and sh*Ddx5* MEFs. UTR, untranslated region. (**D**) Heatmap plots of genes with R-loop signals across the 5 kb window around TSS in the NC, shControl and sh*Ddx5* group. NC, negative control that represents a group without primary antibody in the CUT&Tag assay. (**E**) The genomic distribution of the elevated R-loop CUT&Tag peaks (termed gain peaks) in *Mcm8* KO MEFs relative to WT (left) and sh*Ddx5* group relative to shControl (right). (**F**) Top: Venn diagrams showing the overlaps among shared peaks with gain in R-loop signal upon *Mcm8* KO and *Ddx5* knockdown condition. Bottom: The genomic distribution of the overlapped gain peaks. (**G**) Snapshots of R-loop signals in the representative gene *Cdk4* by genome browser tracks in MEFs. NC (gray), *Mcm8* WT (blue), *Mcm8* KO (green), shControl (purple) and sh*Ddx5* (brown) CUT&Tag data are shown. Source data are available online for this figure.

 © The Author(s)
